# Editorial: Usable and effective digital health for autism care and treatment

**DOI:** 10.3389/fpsyt.2025.1612647

**Published:** 2025-05-29

**Authors:** Wenbing Zhao, Xiongyi Liu, Srinivas Sampalli

**Affiliations:** ^1^ Department of Electrical and Computer Engineering, College of Engineering, Cleveland State University, Cleveland, OH, United States; ^2^ School of Education and Counseling, Levin College of Public Affairs and Education, Cleveland State University, Cleveland, OH, United States; ^3^ Faculty of Computer Science, Dalhousie University, Halifax, NS, Canada

**Keywords:** autism, mobile app, usability, ASD, digital health

According to the US Centers for Disease Control and Prevention, one in every 31 people suffers from autism spectrum disorders (ASD) in 2022, and we have seen an alarming accelerating trend in the prevalence of ASD. Common symptoms of ASD include impairments in cognition, emotional regulation, and social interactions. The impairments usually lead to problematic behavioral issues, which could cause severe disruption to one’s life. While clinical interventions and treatments are available, most people do not have access to such cares due to a variety of reasons, such as shortage of trained clinicians and lack of financial resources to pay for the treatments. The advancement of digital technologies, particularly wearable and mobile technologies, promises to significantly lower the cost of mental health intervention delivery and to greatly expand the access to mental health care.

Most physical illnesses can be rehabilitated within a reasonably short period of time. In contrast, ASD has no known cure and can only be managed. As such, ASD intervention is inevitably long term. Ideally, a digital health intervention for autism should have minimum involvement from the clinician once the initial training with the intervention is completed. This would place significant requirements on the design and implementation of the digital technology as well as the intervention itself. The digital mental health intervention technology must be usable and effective for long-term use. How to achieve this goal is still an open research issue. The goal of this Research Topic is to attract cutting-edge research that would lead to the development of usable and effective digital health intervention technology for long-term autism care and intervention with minimum involvement of trained clinicians. In the end, four original research papers and one mini review were accepted and published in this Research Topic. Two of the papers are about the efficacy of digital social stories. One is about accurately predicting the anxiety level due to sound-induced stress. Another paper is about the how a conversational agent was used by the general public and individuals with autism. Finally, the mini review is about using heart-rate variability biofeedback to mitigate anxiety in ASD.

In the original research paper titled “machine learning model for reproducing subjective sensations and alleviating sound-induced stress in individuals with developmental disorders”, the authors aimed to establish the foundation for personalized support systems for individuals with developmental disorders who experience auditory hypersensitivity by developing deep neural network models that predict changes in subjective sound perception, and determine the modifications needed to sound stimuli to alleviate stress. (Ichikawa et al.) The efficacy of the proposed models were validated experimentally with 28 individuals with developmental disorders, and 29 typically developing individuals. The audio stimuli were produced using an iPad and the experiments were carried out in a soundproof room. The experiments confirmed that the prediction models were able to estimate stress rating with a correlation coefficient of 0.4-0.7 and participants experienced significant reduction in stress ratings in the easing task. Furthermore, the authors observed difference in prediction performance when the models were trained with individual data or group data.

In the original research paper titled “supporting autistic communities through parent-led and child/young person-led digital social story interventions: an exploratory study,” the authors reported a study on using digital social story interventions to support the autistic communities. (Camilleri et al.) The study reported the outcomes of two experiments using a mobile app called SOFA-app as a vehicle to deliver social stories digitally on smartphones. In the first experiment, 17 parents who have young children with ASD participated. These parents used the SOFA-app to deliver social stories to their children. In the second experiment, two children and three adolescents with ASD were recruited. These participants developed their own stories for self-support using the SOFA-app. The results of the experiments demonstrated effectiveness of the interventions.

In the original research paper titled “effective digital support for autism: digital social stories,“ the use of digital social stories as a means of support for autism was also studied. (Camilleri et al.) The authors claimed that this is the largest study on the effectiveness of social stories. Additionally, three specific variables were investigated based on data collected by users of the SOFA-app. The first variable is the closeness-to-goal as rated by parents who use the app to support their ASD children (n=568). The second variable is the children’s comprehension of the social stories assessed by story comprehension questions (n=127). The third variable is the rating of the enjoyability of the social stories as rated by autism children (n=127). Regression analysis was applied to gain deeper insights about the patterns of the three variables. The study found that parental closeness-to-goal ratings for their children were highest for children who were younger and more verbal. Furthermore, older children scored higher in comprehension assessment, and autistic children rated the social stories as more enjoyable. Finally, there is no apparent correlation between closeness-to-goal, comprehension scores and enjoyment ratings.

In the original research paper titled “qualitative analysis of mental health conversational agents messages about autism spectrum disorder: a call for action,” the authors reported the outcome of qualitative analysis of the messages collected from a publicly available mobile app called Wysa, which functions as a conversational agent powered by artificial intelligence technology. (Aghakhani et al.) Both the general public and individuals with ASD have used the agent. The data collected include 1,397 messages from 908 unique users. The messages were dated between June 1, 2021 and July 11, 2022. The study focused on two thematic analyses. One analysis was on user messages that contain predefined keywords related to autism. The other analysis was on messages from those who self-identified as having autism. The study found that the majority of messages had negative valence. Nevertheless, because the app has been extensively used for a wide range of questions regarding autism, the authors concluded that the conversational agent could potentially become a source of support for individuals with autism.

In the mini review titled “Heart rate variability biofeedback to reduce anxiety in autism spectrum disorder – a mini review,” the authors conducted a concise review on studies that focused on using heart-rate variation biofeedback to help individuals with autism to manage anxiety. (Coulter et al.) This review was based on four articles after a systematic literature search and screening. This review identified the characteristics of existing studies, and a few potential issues, such as additional measures are needed to elucidate the links between biofeedback and anxiety for individuals with ASD.

